# Assessing Real World Efficacy, Safety, and 18‐Month Retention Rates of Cannabidiol in Individuals With Drug Resistant Epilepsies

**DOI:** 10.1111/ene.70304

**Published:** 2025-09-18

**Authors:** Nicole Chemaly, Mathieu Kuchenbuch, Emma Losito, Anna Kaminska, Delphine Coste‐Zeitoun, Giulia Barcia, Isabelle Desguerre, Marie Hully, Rima Nabbout

**Affiliations:** ^1^ Department of Pediatric Neurology, Reference Center for Rare Epilepsies Hôpital Necker‐Enfants Malades, Member of ERN EpiCARE Paris France; ^2^ Université de Lorraine, CNRS, IMoPA F‐54000 Nancy France; ^3^ Service de Pédiatrie, Reference Center for Rare Epilepsies, Member of ERN EpiCARE Université de Lorraine, CHRU‐Nancy F‐54000 Nancy France; ^4^ Unit of Pediatric Neurophysiology Necker Enfants Malades Hospital, AP‐HP Paris France; ^5^ Université de Paris Cité Paris France; ^6^ Laboratory of Translational Research for Neurological Disorders INSERM MR1163, Imagine Institute Paris France

**Keywords:** developmental and epileptic encephalopathy, Dravet syndrome, gender differences, Lennox–Gastaut syndrome, off‐label, retention rate, tuberous sclerosis

## Abstract

**Background:**

Highly purified Cannabidiol (CBD, epidiolex) is approved for treating Lennox–Gastaut syndrome (LGS), Dravet syndrome (DS), and tuberous sclerosis (TSC), with off‐label use in drug‐resistant epilepsies. The aim of this study is to evaluate efficacy, safety, and long‐term retention of CBD in a large pediatric population.

**Methods:**

We prospectively included all consecutive patients initiating CBD for drug‐resistant epilepsy at our pediatric tertiary center from 2019 to 2021. Efficacy and safety were assessed via parental questionnaires on seizure and non‐seizure outcomes at 1, 2, and 6 months, comparing each item to prior clinic visits. Retention rate was evaluated at 18 months.

**Results:**

We included 103 patients (mean age 11.2 years), with 47% receiving CBD for label‐approved syndromes (23 DS, 15 LGS, 10 TSC) and 53% off‐label. At 1 month, 54% of caregivers reported reduced seizure frequency and duration, with a positive overall impression in 62%. By months 2–6, 48% maintained seizure improvement, alongside sustained gains in communication (60%), alertness (54%), and motor skills (44%). Retention rates were 97% at 1 month, 90% at 2 months, 82% at 6 months, 66% at 12 months, and 55% at 18 months. Off‐label use (*p* = 0.02), male gender (*p* = 0.049), and older age at initiation (*p* = 0.03) were associated with higher retention.

**Conclusions:**

CBD reduced seizures and improved non seizures' morbidities in drug‐resistant epilepsy. Strong 18‐month retention rate, including off‐label use, highlights efficacy and tolerability, underscoring the need for further real‐world data in various rare epilepsy syndromes beyond its market approval.

## Graphical abstract

Prospective single‐centre study of 103 children with drug‐resistant epilepsy treated with purified cannabidiol: 47% on‐label conditions and 53% off‐label. CBD was generally well tolerated, with few major side effects, and both seizure‐related and non‐seizure‐related benefits could require up to 6 months to emerge. Retention was 56% at 18 months, declining most in girls, younger patients, and those with on‐label conditions.

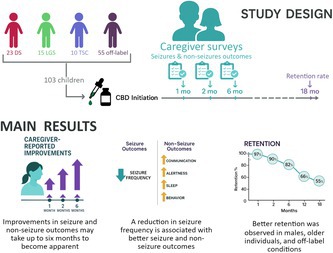

## 1 Introduction

Currently, more than 30 anti‐seizure medications (ASMs) have been approved by the Food and Drug Administration [1]. The primary goal of ASMs is to modulate abnormal electrical activity in the brain to prevent epileptic seizures. The choice of a specific ASM is guided by the clinician's decision‐making process based on consensus guidelines and literature data, market authorization, spectrum of anti‐seizure activity (narrow or broad), tolerance profile, adverse events, patient's characteristics such as gender, age, epilepsy syndrome, and comorbidities, and last on his own experience [2]. This process allows clinicians to adopt a patient‐tailored approach, aiming for efficiency, an optimal balance between efficacy and tolerability.

However, achieving this balance becomes especially challenging in patients with developmental and epileptic encephalopathies (DEE). Indeed, in this group of epilepsy syndromes, epilepsy is difficult to treat and patients' needs extend beyond seizures' management. DEE is often associated with a range of comorbidities, including behavioral and psychiatric disorders, autism spectrum disorders, communication difficulties, sleep and eating disturbances, and gait abnormalities [3]. While the goal in pediatric epilepsy management is often to achieve complete seizure freedom, in DEEs, optimal management may instead focus on achieving the optimal seizure reduction, to minimize their impact, making them more manageable for the patient and the family's daily life, while avoiding ASM‐related adverse events.

In recent years, four out of the five most recent FDA‐approved treatments, fenfluramine, cannabidiol (CBD), everolimus, and stiripentol—have been specifically indicated for this challenging population with high unmet needs. These approvals were based on randomized clinical trials, with primary endpoints focusing on efficacy, typically measured by the reduction in seizure frequency, such as the responder rate (defined as the percentage of patients achieving at least a 50% reduction in total seizure frequency) [4, 5, 6, 7, 8, 9, 10, 11, 12, 13, 14, 15]. Secondary endpoints focused on safety, particularly the incidence of adverse events (AEs), including serious AEs and withdrawal rates due to AEs.

A study involving caregivers of individuals with Dravet syndrome (DS) identified that improvements in sleep, communication, behavior, daily activities, motor skills, and language development are considered more important treatment targets than changes in seizure characteristics, particularly seizure frequency [3]. This finding highlights the importance of patient‐reported outcome measures, which, in pediatric cases with intellectual disabilities, often rely on caregiver observations. Indeed, parents and caregivers play a crucial role in assessing the overall impact of treatment on the child's quality of life, encompassing both seizure and non‐seizure domains. Their perspectives provide valuable insights that often align with formalized assessments [16, 17, 18].

In this context, we conducted a prospective study to evaluate the real‐world effectiveness and safety of CBD in children with drug‐resistant epilepsy regardless of market authorization. Our aim was to assess both seizure and non‐seizure outcomes at 1, 2, and 6 months. Finally, we analyzed retention rates up to 18 months and examined factors associated with retention.

## 2 Methods

### 2.1 Participants

We prospectively included consecutive children with drug‐resistant epilepsy who started CBD as an add‐on therapy between 2019 and 2021, either for a condition covered by market authorization (DS, LGS, and TSC) or outside this framework. The study was approved by the local ethics committee. Parents received detailed information from the referring pediatric neurologist (RN and NC) and provided informed consent before completing questionnaires.

### 2.2 Methods

We developed a survey in collaboration with families and experts to assess the impact of adding Cannabidiol (CBD) on both seizure and non‐seizure outcomes. The survey was based on previous studies and literature data [3, 19, 20]. It used a seven‐point Likert scale ranging from −3 (major aggravation) to 3 (major improvement), with 0 indicating no change. Seizure‐related outcomes assessed included frequency, duration, recovery quality, use of rescue therapies, emergency calls, emergency room visits, hospital admission to continuous or intensive care, and change in sensitivity to trigger factors such as fever and photic sensitivity. Additionally, non‐seizure outcomes were evaluated, including general global impression of the caregiver, communication/interaction, language, alertness, behavior, motor skills, sleep, and appetite. To track changes over time, we asked caregivers to note the changes from the last visit.

CBD was introduced following our previously established slow titration protocol to enhance tolerability [19]. The dose increased by 2.5 mg/kg/day weekly, reaching 10 mg/kg/day at 1 month, then by 2 mg/kg/day weekly based on efficacy and tolerability.

### 2.3 Statistical Analysis

Descriptive statistics were reported as number (percentage) and median [25th–75th percentile]. Descriptive analyses included demographic data (gender, date of birth), epilepsy‐related data (age at first seizure, epileptic condition [DS, LGS, TSC, other]) and CBD use (age at CBD introduction, dosage, adjunctive medications, discontinuation status, and if discontinued, the reason and date).

We performed the Kruskal–Wallis test on Likert scale responses to assess the relationship between seizure frequency changes and both seizure‐related and non‐seizure outcomes across groups showing improvement, no change, or worsening. Likert‐scale evaluated outcomes were also compared between the group with vs. without clobazam co‐medication using Wilcoxon rank‐sum. To account for multiple testing, Bonferroni correction was applied.

Finally, to identify factors associated with CBD withdrawal before 18 months, we used univariate Cox proportional hazards models. Initially, we tested the factors of age, gender, presence or absence of clobazam, condition category (DS, LGS, TSC, and others), and CBD dosage. Factors with a *p* value < 0.2 were subsequently included in a multivariate Cox proportional hazards model. A *p* value < 0.05 was considered statistically significant, and a *p* value < 0.1 was considered a trend. Statistical analyses were conducted using R software.

## 3 Results

### 3.1 Population

A total of 103 individuals were enrolled in this study (Table 1). The sex ratio was 1.02. Epilepsy onset was at a median age of 6 [3–18] months. Among participants, 47% had a condition targeted by CBD market authorization of the EMA (23 individuals with DS, 15 with LGS, 10 with TSC) whereas 53.4% received CBD in off‐label conditions (*n* = 55, Table S1). CBD was introduced at 11 [6.9–15.1] years as an add‐on to 3 [2, 3] ASMs, including clobazam in 48.5% (*n* = 50). All patients had a slow titration protocol, reaching 10 mg/kg/day at M1. The maximal dosage of CBD was 14.4 [11.5–16.3] mg/kg/day. Surveys about the impact of CBD on seizures and non‐seizure outcomes were completed by 82%, 77.4%, and 61.9% of individuals still under CBD at M1 (82 out of 100 individuals), M2 (72 out of 93) and M6 (52 out of 84), respectively.

**TABLE 1:** Basic demographic and clinical characteristic of individuals enrolled in this study and comparison of subpopulations of individual with DS, LGS, TSC and other conditions.

		Total	Dravet	LGS	TSC	Other	*p*
*N*		103	23 (22.3%)	15 (14.5%)	10 (9.7%)	55 (53.4%)	
Gender (F/M)		51/52	13/10	5/10	6/4	27/28	ns
Seizure onset (months)		6 [3–18]	5 [3–6.25]	10 [5–36]	2 [1.25–2.75] *	10 [4–36]	< 10^−4^
Age at CBD introduction (years)		11 [6.9–15.1]	8.61 [6.1–12.4]	11.4 [7.7–14.8]	7.98 [5.5–10.9]	11.98 [8.1–15.7]	ns
Number of ASM at CBD introduction		3 [2–3]	3 [2–3]	3 [3–3.5]	2 [2–2.75]	2 [2–3]	ns
Presence of clobazam		50 (48.5%)	17 (73.9%) *	6 (40%)	5 (50%)	22 (40%)	0.04
CBD Max dosage (mg/kg/day)		14.4 [11.5–16.3]	14.5 [14.3–16]	11.6 [10–15.5] *	13.8 [10–16]	14.7 [11.8–16.8]	< 10^−4^

The comparisons across four subgroups‐individuals with DS, TSC, LGS, and the group of off‐label etiologies‐showed no significant difference in terms of gender, age at CBD introduction, and number of ASMs before CBD. However, significant differences were observed in seizure onset (*p* < 10^−4^), presence of clobazam in the comedication (*p* = 0.04) and maximum dosage of CBD (*p* < 10^−4^) among subgroups. Pairwise comparisons identified individuals with TSC as having the earliest onset of seizures (*p* < 0.05 for all pairwise comparisons). Individuals with DS had the highest proportion of clobazam use in their ASM regimen (73.9%, *p* = 0.008). Individuals with LGS received the lowest target dose of CBD (11.6 [10–15.5] mg/kg/day, *p* < 10^−4^) compared to other subgroups. The off‐label etiologies subgroup did not differ significantly from the other groups in gender, age at CBD initiation, or number of ASMs in their regimen.

### 3.2 Evaluation of CBD Impact on Seizure and Daily Life Aspects by Caregivers

**FIGURE 1:**
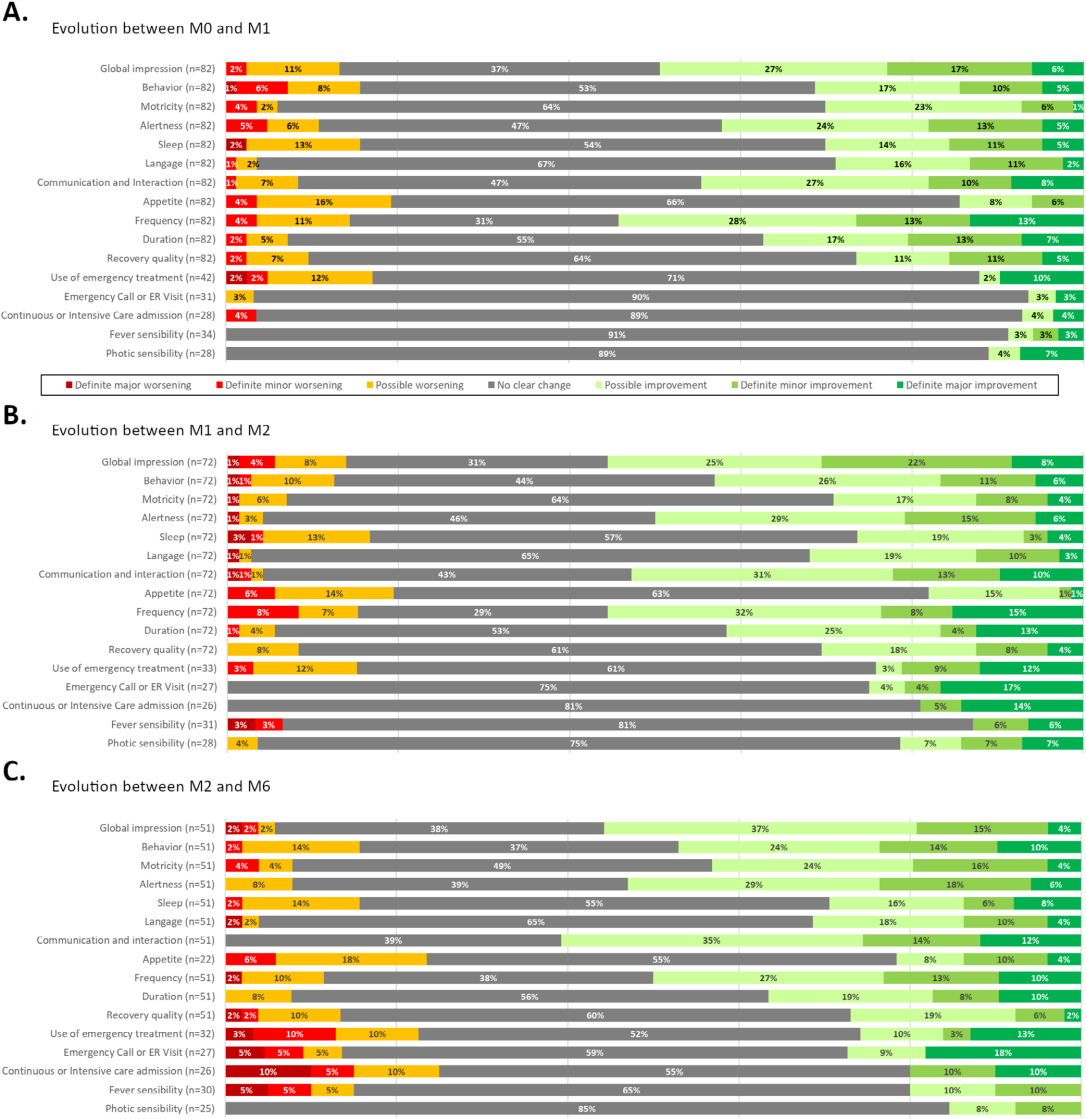
Changes in seizure and/or daily life characteristics observed by caregivers from M0 to M1, M1 to M2, and M2 to M6.

One month after CBD introduction, 54% of caregivers reported an improvement in seizure frequency (*n* = 44/82, Figure 1). Half of them also reported an improved global impression (*n* = 41), particularly in communication and interaction skills (45%, *n* = 37), as well as alertness (42%, *n* = 35). The duration of seizures also seemed to be reduced (37%, *n* = 30). About one‐quarter of individuals (*n* = 18) experienced major improvements in 3 [1.25–4] domains, mainly seizure frequency (*n* = 11), communication and interaction (*n* = 7) and seizure duration (*n* = 6). Conversely, a worsening of 1 [1–3.5] seizure or non‐seizure outcomes was reported in 45% of individuals (*n* = 37). The most identified adverse events were decrease of appetite (20%, *n* = 16), use of emergency treatment (16%, *n* = 13), and sleep disturbances and behavior changes, both at 15% (*n* = 12). Major worsening occurred in 5% of individuals (*n* = 4), mainly in sleep (*n* = 2), use of emergency treatment (*n* = 2) and behavior (*n* = 1).

Two months after the introduction, caregivers continued to report improvements compared to Month 1, particularly in seizure frequency (55%, *n* = 40/72), global impression (55%, *n* = 40), communication and interactions (54%, *n* = 39), alertness (50%, *n* = 31) and seizure duration (36%, *n* = 26). Additionally, families also reported an improvement in behavior (43%, *n* = 31). About a quarter of individuals (*n* = 18) experienced major improvements in 3 [2‐5] aspects of their seizure and/or non‐seizure outcomes, primarily in seizure frequency (*n* = 11), seizure duration (*n* = 9), communication and interactions (*n* = 7) and global impression (*n* = 6). Half of the individuals (*n* = 36) reported a worsening in 2 [1–3] domains, mainly appetite (19%, *n* = 14), sleep (17, *n* = 12%), seizure frequency (15%, *n* = 11) and use of rescue therapy (15%, *n* = 11). Major worsening was identified in 7% of individuals (*n* = 4), mainly on sleep (*n* = 2).

Between M2 and M6, the proportion of individuals reporting improved seizure frequency slightly decreased (48%, *n* = 24/52). However, global impression tended to improve during this period for 58% of individuals (*n* = 29), along with communication and interaction (60%, *n* = 30), alertness (54%, *n* = 27), behavior (46%, *n* = 23) and motricity (44%, *n* = 22). Nearly one‐third of individuals experienced major improvement (*n* = 15) in 4 [2–6.25] aspects of their seizure and/or non‐seizure outcomes, mainly language (*n* = 9), alertness (*n* = 9) and motricity (*n* = 8). Worsening of 3 [1–6] of their characteristics, mainly appetite (22%, *n* = 11), sleep (16%, *n* = 8) and behavior (16%, *n* = 8), was reported by two‐thirds of individuals between M2 and M6 (*n* = 33). However, major worsening was identified in only 2% (*n* = 1) concerning seizure frequency.

**FIGURE 2:**
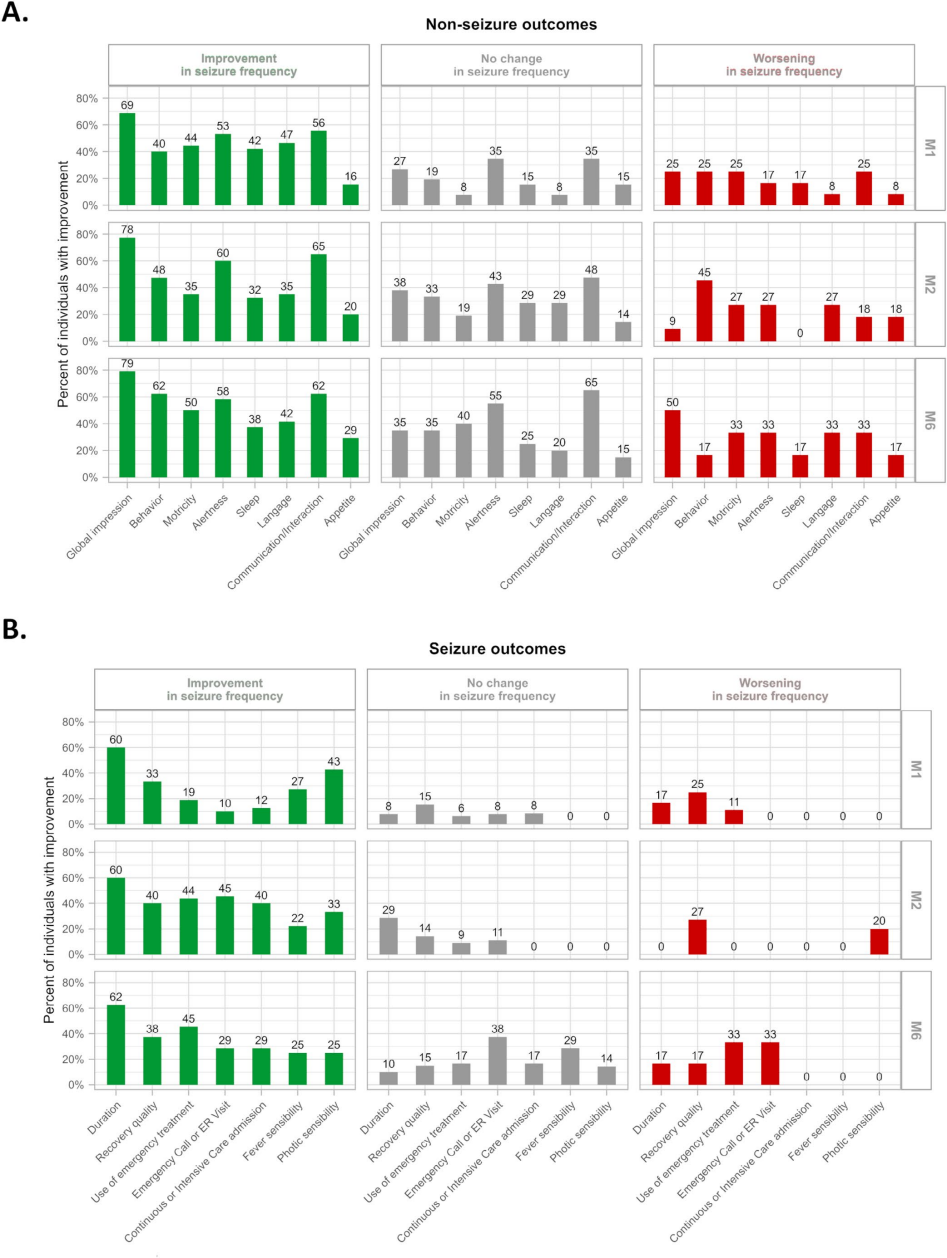
Relationship between caregiver‐observed seizure frequency response and other seizure and/or daily life characteristics from M0 to M1, M1 to M2, and M2 to M6.

Over time, both seizure‐related and non‐seizure outcomes showed progressive and sustained improvement with CBD treatment. At 1 month, more than half of caregivers reported reduced seizure frequency, along with improvements in communication, alertness, and overall well‐being. By 2 months, these positive effects persisted, with additional progress observed in behavior. Between Month 2 and 6, seizure‐related outcomes slightly declined, while non‐seizure outcomes such as communication, alertness, behavior, and motor skills continued to improve. Nearly one‐third of individuals experienced major improvements across multiple domains, particularly in language, alertness, and motor skills. The comparison of seizure and non‐seizure outcomes between individuals with improved, unchanged, or worsened seizure frequency was not homogeneous (*p* < 10^−4^, Figure 2). Indeed, individuals with improved seizure frequency also exhibited better seizure and non‐seizure outcomes, while those with unchanged or worsened seizure frequency showed less favorable progress. Comparison of outcomes between patients with and without clobazam co‐medication revealed no statistically significant differences after Bonferroni correction. Nominal differences were observed for appetite at M2 and sleep at M6 (*p* = 0.018), but they did not reach statistical significance after correction (Figure S1).

### 3.3 CBD Retention Rates

**FIGURE 3:**
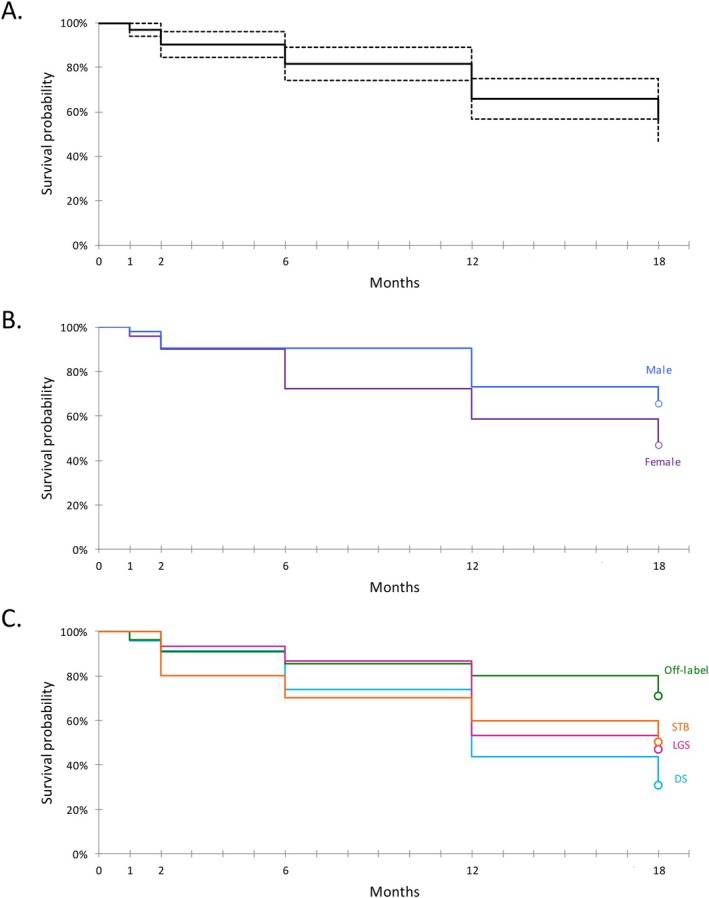
Survival Curves of CBD Retention Rate (A) and Influence of Gender (B) and Conditions (C) on Retention Rate.

CBD retention rates were 97% (*n* = 100), 90% (*n* = 93), 82% (*n* = 84), 66% (*n* = 68), and 56% (*n* = 58) at 1, 2, 6, 12, and 18 months, respectively (Figure 3A). Cox proportional hazards model revealed significant effects of sex (*p* = 0.049), age of CBD introduction (*p* = 0.03) and epilepsy syndrome category (*p* = 0.02) on retention rates. Specifically, males had higher retention rates than females (Figure 3B), younger age at CBD introduction was associated with lower retention rates, and off‐label conditions had higher retention rates than conditions covered by marketing authorization (Figure 3C). Multivariate analysis confirmed that these effects were independent (age: *p* = 0.058, gender: p = 0.02, off‐label conditions: *p* = 0.005). Comparisons between male and female subpopulations did not reveal significant differences in the number of ASMs, CBD dose, or clobazam add‐on.

## 4 Discussion

Our study is a prospective investigation of CBD use in a real‐world setting at a tertiary center for rare epilepsy. Several key points can be highlighted. First, over 50% of CBD use in our center was for off‐label conditions. Second, CBD demonstrated good tolerability, with few major side effects. Third, its efficacy, particularly in reducing seizures and improving quality of life, may take up to 6 months to become evident. Lastly, the 18‐month retention rate was 56%, with higher rates observed in older patients, males, and those using CBD for off‐label conditions.

Off‐label use of CBD in children with drug‐resistant epilepsy is well‐documented, with real‐world data indicating usage rates between 13% and 66% [21, 22, 23, 24, 25]. Our findings place us at the higher end of this range. While randomized controlled trials (RCTs) remain the gold standard, observational studies provide invaluable insights into treatment effectiveness in clinical practice, particularly for identifying populations, such as those with *SYNGAP1*, who may benefit from CBD and are unlikely to be included in RCT due to recruitment challenges [26]. Moreover, our real‐world data confirm that individuals who exhibit a good response in seizure frequency reduction often also show improvements in non‐seizure outcomes, such as emotional and behavioral functioning [20]. We hypothesize that better seizure control contributes to improvements in the epileptic components of DEE. Understanding the extent of off‐label use in these populations can help refine clinical practice and guide treatment decisions for individuals with limited therapeutic options.

Given the known pharmacokinetic interaction between CBD and clobazam, CBD increasing the levels of Nor‐CLB [27, 28, 29], we performed stratified analyses to evaluate the potential impact of clobazam co‐medication. Although nearly half of the cohort received clobazam, caregiver‐reported outcomes showed no statistically significant difference after Bonferroni correction. Our findings suggest that clobazam co‐medication may not substantially influence the perceived clinical benefit of CBD in this cohort, though further studies with larger samples and pharmacokinetic evaluation are needed. In addition, therapeutic drug monitoring (TDM) was not implemented in this study. While logistically difficult in routine practice, TDM may help better define dose–response relationships and reduce interindividual variability, particularly in patients receiving polytherapy, especially with clobazam, valproate, stiripentol, or fenfluramine. Recent studies highlighted substantial interindividual variability in plasma concentrations of CBD and its major metabolites, particularly 7‐OH‐CBD and 7‐COOH‐CBD [30, 31]. This may underscore the role of close clinical monitoring and the integration of TDM to support individualized CBD treatment strategies and improve therapeutic precision.

In our study, we observed that improvement, mainly of non‐seizure outcomes, could emerge up to 6 months after treatment initiation. This medium‐term window may be crucial for accurately assessing CBD's efficiency. Discontinuing treatment before 6 months may deprive highly pharmacoresistant individuals, who have already undergone multiple ASM trials, of a potential therapeutic benefit. Indeed, some individuals exhibited consistent benefits between 2 and 6 months after CBD initiation. This delayed response underscores the need for patients' and families' education and careful evaluation of treatment impact and highlights the importance of longer‐term assessments to fully capture the therapeutic potential of CBD, particularly in complex and pharmacoresistant epilepsies. Our study extends the evaluation of the efficiency beyond the 14 weeks of the RCTs upon which the drug has been approved [4, 5, 8, 12, 32]. Furthermore, our cohort experienced good tolerability overall, with a few major side effects. The most reported adverse effects included changes in appetite, behavioral disturbances, and, to a lesser extent, sleep issues. Although sleep disturbances are less documented, they can significantly impact quality of life and should not be overlooked in clinical assessments. Importantly, we observed no significant hepatic adverse events both biologically (liver enzyme activity) and clinically, indicating that CBD can be administered safely. The median dose in our population was 14 mg/kg/day, lower than the target dose of 20 mg/kg/day in LGS and DS and 25 mg/kg/day in TSC [12, 21, 33].

Importantly, clinicians cannot accurately evaluate the efficacy and safety of antiseizure medications (ASMs) on both seizure‐related and non‐seizure outcomes without the active involvement of caregivers. Engaging parents in the evaluation process fosters a meaningful partnership that is essential for assessing children with DEEs, particularly in areas where standardized scoring systems may be unavailable, difficult to implement, or time‐consuming. It is important to note that seizures and non‐seizure symptoms often fluctuate significantly, and caregivers' observations provide valuable insights into these variations, which may not always be captured during the limited time of the clinical visits. Additionally, clinical assessments in this population are often compromised by the challenges of testing outside the child's usual environment, where difficulties can be exaggerated.

Caregiver‐reported questionnaires can be as informative as clinical assessments, providing critical data that reflect the child's daily experiences. However, this raises the question of which treatment aspects should be prioritized for improvement; caregivers' impressions alone are insufficient. This underscores the need for collaborative work with families to establish patient‐centered treatment goals. Relying solely on broad assessment scales may lack the granularity needed for meaningful comparisons within patient groups. Training parents in structured evaluation methods can help minimize subjectivity and improve the reliability of caregivers‐reported data. By integrating parental input into the evaluation process, clinicians can gain a more comprehensive understanding of a child's needs, allowing tailored treatment approaches that ultimately enhance care quality and outcomes for children with drug‐resistant epilepsy. In future studies, caregiver‐reported impressions could be usefully complemented by validated structured tools such as the Aberrant Behavior Checklist or the PedsQL Epilepsy Module, which require less than 15 min to complete [34]. These instruments would improve the objectivity of reported outcomes while remaining feasible for families and would facilitate comparisons across studies and populations.

Our 18‐month retention rate of 56% aligns with findings from the open‐label extension program and other real‐world data cohorts [22, 23, 24]. This rate reflects the balance between CBD treatment efficacy and safety. However, unlike previous studies that did not investigate factors affecting retention, we identified that off‐label use, male gender, and older age at treatment initiation may be positively associated with higher retention rates. We were particularly surprised by the effect of gender, as we did not expect this outcome in our population. We investigated potential cofactors to explain this difference but found no significant variations in terms of the number of treatments, dosages, the presence or absence of clobazam, or etiology. In the study by Georgieva et al., females made up 64% of the discontinuation group, while only 49% of those who continued CBD were female, mirroring our findings [24]. This gender difference is further supported by preclinical studies in healthy volunteers, which have shown that females exhibit greater relative exposure to CBD metabolites in plasma over time, as well as gender‐specific variations in cannabinoid pharmacokinetics. Specifically, females demonstrated higher Cmax and AUC values than males. Similar trends were observed in preclinical studies, where females had a 2.25‐fold higher Cmax and a 1.97‐fold higher AUC for 7‐COOH‐CBD after normalizing for body weight by Day 4 of administration [35, 36]. Female subjects had a normalized Cmax of 39.81 ng/mL/kg and AUC of 217.70 ng/mL.h/kg, nearly double that of males. Additionally, a significant correlation between 7‐OH‐CBD AUC and body weight was observed in females. Authors have suggested that sex differences in UGT activity, such as slower UGT2B15 metabolism in females, may contribute to these pharmacokinetic differences [37]. Our study is among the first to highlight a gender effect in the long‐term retention of CBD treatment. While the mechanisms remain uncertain, emerging pharmacokinetic data suggest that females may have greater exposure to active metabolites, possibly affecting tolerability. This observation, consistent with previous real‐world and preclinical findings, adds to the ongoing debate regarding sex differences in CBD efficacy and metabolism [38]. Further sex‐specific studies are needed to clarify these differences and support more personalized approaches to CBD therapy. Such research should aim to elucidate the pharmacokinetic and pharmacodynamic mechanisms underlying sex‐related variability, assess their impact on both efficacy and tolerability, and determine whether dose adjustments or monitoring strategies should differ between males and females in clinical practice.

This study, although prospective and including a relatively large number of patients, has some limitations. First, as it was conducted in a single tertiary center, the findings may not be fully generalizable to other settings with different patient populations. Additionally, the significant proportion of off‐label CBD use introduces variability in treatment protocols, which could influence retention rates and outcomes. While caregiver‐reported outcomes are valuable, they may introduce some subjectivity, particularly in assessing non‐seizure outcomes. Lastly, the absence of a control group limits direct comparison of CBD effectiveness to other treatments.

## 5 Conclusion

Our study provides valuable insights into the real‐world data on the role of CBD in the treatment of pediatric drug‐resistant epilepsies, reinforcing its potential beyond currently approved indications, highlighting both the efficacy and possible factors that influence its long‐term retention. CBD demonstrated good tolerability, with few major side effects. Notably, its therapeutic effects, particularly in seizure and non‐seizure outcomes, may take up to 6 months to manifest, emphasizing the importance of sustained treatment before considering discontinuation. Our findings also emphasize the critical role of caregivers' involvement in treatment evaluation, especially for pediatric patients with intellectual disability. By integrating parental input, clinicians can better assess a child's response to CBD beyond seizure control, capturing more comprehensive improvements in emotional and behavioral functioning. As the role of CBD in managing drug‐resistant epilepsy continues to evolve, our study contributes to a deeper understanding of how to optimize treatment strategies, ensuring that patients receive the full benefit of this promising therapy.

## Author Contributions

**Nicole Chemaly:** conceptualization, writing – review and editing, data curation, writing – original draft, investigation, validation, methodology. **Mathieu Kuchenbuch:** formal analysis, writing – original draft, visualization, writing – review and editing, investigation, software, data curation, methodology. **Emma Losito:** writing – review and editing, investigation. **Anna Kaminska:** writing – review and editing, investigation. **Delphine Coste‐Zeitoun:** writing – review and editing. **Giulia Barcia:** writing – review and editing. **Isabelle Desguerre:** writing – review and editing. **Marie Hully:** writing – review and editing. **Rima Nabbout:** writing – review and editing, conceptualization, methodology, investigation, supervision, project administration, validation.

## Conflicts of Interest

The authors declare no conflicts of interest.

## Supporting information


**Table S1:** Detailed demographic and clinical characteristics of patients receiving off‐label CBD add‐on. **Figure S1:** Outcomes across clinical domains and timepoints comparing patients with and without clobazam co‐medication.

## Data Availability

The data that support the findings of this study are available on request from the corresponding author. The data are not publicly available due to privacy or ethical restrictions.
