# Supplementary material for: Assessing Real World Efficacy, Safety, and 18‐Month Retention Rates of Cannabidiol in Individuals With Drug Resistant Epilepsies
Source: Eur J Neurol. 2025 Sep 18;32(9):e70304. doi: 10.1111/ene.70304 (PMC12446575; doi:10.1111/ene.70304)
Supplement: Supplementary file 1 — Table S1: Detailed demographic and clinical characteristics of patients receiving off‐label CBD add‐on. Figure S1: Outcomes across clinical domains and timepoints comparing patients with and without clobazam co‐medication. [file ENE-32-e70304-s001.docx]

| **Gender** | **Age at CBD initiation  (years)** | **Epilepsy syndrome** | **Seizure type** | **Pathogenic  variant / etiology** | **Retention (months)** | **ASM comedication** |
| --- | --- | --- | --- | --- | --- | --- |
| M | 10,1 | DEE | Spasms, atypical absences | ? | 18 | TPM,RFM |
| F | 4,7 | EIMFS | Focal seizures | *SCN8A* | 6 | VGB, CBZ, PHT |
| M | 3,8 | GGE | Atypical absence | *SYNGAP1* | 18 | CLB |
| M | 4,6 | EIMFS | Focal seizures | *KCNT1* | 2 | CLB |
| F | 8,4 | EMAtS | Atonic, myoclonic seizures | *SYNGAP1* | 18 | LTG, ZNS |
| M | 6,4 | GGE | Atypical absence, myoclonic seizures | *SYNGAP1* | 18 | VPA, LTG |
| F | 19,6 | DEE | Generalized tonic clonic seizures | *SCN2A* | 18 | ZNS, CLB, VPA |
| M | 1,7 | EIMFS | Focal seizures | *KCNT1* | 18 | VGB, CBZ |
| F | 16,7 | DEE | Atypical absences | *GRIN2A* | 18 | VPA, STM, CLB |
| F | 7,4 | DEE | Tonic seizures, atypical absences | *NBEA* | 18 | VPA, LTG, CLB, RFM |
| F | 14,7 | GGE | Atypical absence, tonic-clonic seizures | *SETD1B* | 18 | VPA, LTG, CLB |
| M | 12,9 | DEE | Tonic seizures, spasms, atypical absences | Herpes simplex meningoencephalitis | 18 | CBD, CBZ |
| M | 7,8 | DEE | Reflex tonic seizures | Clastic lesion | 18 | LEV, RFM |
| M | 19,8 | DEE | Generalized tonic and tonic-clonic seizures | ? | 18 | VPA, LTG, CLB |
| M | 20,6 | DEE | Generalized tonic-clonic seizures | *OCRL1* | 18 | VPA, VGB |
| F | 19,4 | GGE | Atypical absences with photosentivity | ? | 1 | VPA |
| F | 16,5 | Focal epilepsy | Focal seizures | ? | 18 | VPA, OXC, CLB |
| F | 13,8 | GGE | Generalized tonic, tonic-clonic, and atypical absence seizures | *GRIN2A* | 12 | VPA, CBZ, PER, CLZ |
| F | 10,4 | GGE | Atypical absences | *SYNGAP1* | 18 | VPA, ETX |
| F | 10,3 | GGE | Absence seizures | ? | 18 | VPA, ETX |
| M | 8,8 | PME | Myoclonic, generalized tonic seizures | ? | 18 | VPA, LTG, CLB, LEV |
| M | 9,6 | DEE | Generalizes tonic clonic seizures | ? | 18 | VPA, TPM, LTG, CLN |
| F | 13,6 | DEE | Focal seizures | *GABRB3* | 18 | LCM, TPM |
| M | 11,5 | DEE | Generalized tonic-clonic seizures | ? | 18 | VPA, TPM, FFA |
| F | 18,4 | DEE | Clusters of generalized tonic clonic seizures | *SCN1A* | 18 | VPA, TPM, CLB |
| F | 3,8 | IESS | Spasms, focal seizures | ? | 2 | LCM |
| F | 14,2 | GGE | Myoclonic seizures | ? | 18 | PER |
| F | 12,0 | GGE | Generalized tonic clonic seizures | *CHD2* | 1 | LVT |
| M | 15,6 | DEE | Spasms seizures | ? | 18 | VPA, LTG, TPM |
| M | 9,9 | DEE | Generalized tonic clonic seizures, tonic seizures | *IQSEC2* | 18 | VGB, CBZ |
| F | 11,2 | EIMFS | Focal seizures | *KCNT1* | 12 | CLZ |
| M | 18,1 | DEE | Focal to bilateral tonic clonic seizures | ? | 18 | LTG |
| F | 15,6 | DEE | Generalized tonic seizures | ? | 18 | VPA, LTG |
| M | 19,2 | DEE | Focal seizures | *SCN8A* | 18 | VPA, CBZ, CLB |
| F | 15,7 | DEE | Generalized tonic-clonic seizures | ? | 6 | TPM, LTG, RFM |
| M | 8,7 | DEE | Atypical absences, generalized tonic clonic seizures | ? | 18 | LEV, CLZ |
| M | 2,4 | DEE | spasms | Clastic lesion | 18 | CBZ, VGB |
| F | 15,5 | DEE | Focal seizures | *CNTNAP2* | 2 | VPA, LTG, CBZ |
| F | 12,8 | DEE | Tonic seizures | *KCNB1* | 18 | VPA, LTG |
| F | 14,8 | DEE | Atypical absences | *CACNA1A* | 18 | CLZ |
| M | 10,4 | DEE | Generalized tonic seizures | *ATP1A3* | 12 | CLB, VPA, CLZ, LTG |
| M | 16,8 | DEE | Generalized tonic seizures | Infectious meningitis | 18 | VPA, LTG, RFM |
| M | 6,7 | DEE | Spasms, generalized tonic clonic seizures | *IQSEC2* | 18 | VGB, TPM |
| M | 11,8 | DEE | Generalized tonic, tonic-clonic seizures | ? | 18 | LEV, RFM, CLB |
| F | 15,7 | DEE | Generalized tonic clonic seizure | ? | 18 | CBD, VPA, CLZ, PER, CBZ |
| M | 13,7 | GEFS+ | Generalized tonic clonic seizure | *SCN1A* | 18 | VPA, CLB |
| M | 5,5 | DEE | Generalized tonic clonic seizure | *TESC2* | 18 | VGB |
| F | 12,3 | GEFS+ | Generalized tonic clonic seizure | ? | 12 | VPA, STP, CLB |
| F | 22,4 | GEFS+ | Generalized tonic clonic seizure | *SCN1A* | 2 | VPA, CLB, TPM |
| M | 8,8 | DEE | spasms | ? | 6 | LTG, VPA |
| F | 14,4 | DEE | Atypical absences | ? | 18 | STM, ETX |
| F | 2,2 | EIMFS | Focal seizures | *KCNT1* | 18 | VPA, STP |
| M | 2,7 | IESS | Spasm seizures | Infectious meningitis | 1 | LEV, VGB, TPM |
| M | 19,8 | DEE | Tonic seizures | *GRIN2D* | 18 | VPA, LTG, RFM, CBD |
| M | 6,3 | EMAtS | Atonic, myoclonic seizures | ? | 1 | VPA, LTG, LVT, CLZ |

**Supplementary Table 1**. **Detailed demographic and clinical characteristics of patients receiving off-label CBD add-on.**

Abbreviations: CBZ, carbamazepine; CBD, cannabidiol; CLB, clobazam; CLN, clonidine; CLZ, clonazepam; ETX, ethosuximide; FFA, fenfluramine; LCM, lacosamide; LEV, levetiracetam; LTG, lamotrigine; LVT, lacosamide; OXC, oxcarbazepine; PER, perampanel; PHT, phenytoin; RFM, rufinamide; STM, sultiame; STP, stiripentol; TPM, topiramate; VGB, vigabatrin; VPA, valproic acid.


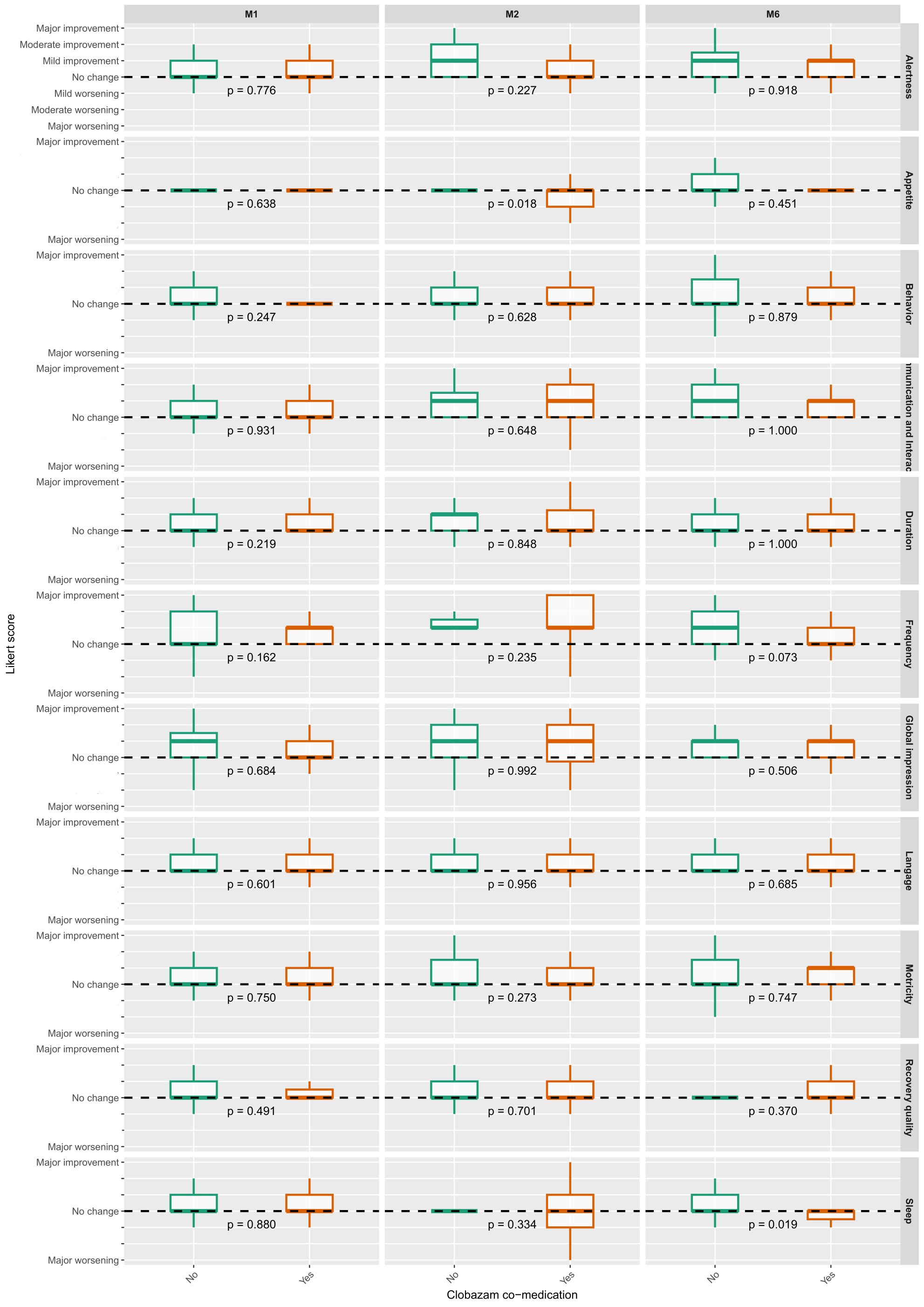


**Supplementary Figure: Outcomes across clinical domains and timepoints comparing patients with and without clobazam co-medication.**

P-values are derived from Wilcoxon rank-sum tests and adjusted for multiple comparisons using the Bonferroni method. A corrected p-value < 0.0033 was considered significant, based on 15 independent comparisons.
